# Validation of the Arabic and French Versions of a Knowledge, Attitudes and Practices (KAP) Questionnaire on Tranquilizer Misuse

**DOI:** 10.3390/ijerph182111144

**Published:** 2021-10-23

**Authors:** Narmeen Mallah, Rubén Rodríguez-Cano, Danielle A. Badro, Adolfo Figueiras, Francisco Caamaño-Isorna, Bahi Takkouche

**Affiliations:** 1 Department of Preventive Medicine, University of Santiago de Compostela, 15782 Santiago de Compostela, Spain; narmeen.mallah@usc.es (N.M.); adolfo.figueiras@usc.es (A.F.); bahi.takkouche@usc.es (B.T.); 2 Centro de Investigación Biomédica en Red de Epidemiología y Salud Pública (CIBER-ESP), 28029 Madrid, Spain; 3 Health Research Institute of Santiago de Compostela (IDIS), 15706 Santiago de Compostela, Spain; 4 PROMENTA Research Center, Department of Psychology, University of Oslo, 0315 Oslo, Norway; r.r.cano@psykologi.uio.no; 5 Faculty of Health Sciences, American University of Science and Technology, Beirut 1100, Lebanon; dbadro@aust.edu.lb; 6 Institut National de Santé Publique, Epidémiologie Clinique et Toxicologie (INSPECT-LB), Beirut 1100, Lebanon

**Keywords:** Arabic, French, knowledge, attitudes and practices, misuse, questionnaire validation, tranquilizers

## Abstract

Tranquilizer misuse is an emerging international public health concern. The psychosocial determinants of this misuse remain understudied. Instruments to measure the Knowledge, Attitudes and Practices (KAP) of tranquilizer misuse are unavailable, except for a recently published questionnaire validated in the Spanish language. We translated the KAP questionnaire into Arabic and French, adapted it and undertook a complete validation procedure in the general adult population in Lebanon. The content validity indicators were good: item content validity index ranged between 0.89 and 1.00, the content validity index scale average was ≥0.95 and the modified Kappa statistic for each of the KAP items was equal to I-CVI. The intra-class correlation coefficient values (*n* = 100) were ≥0.62 for all Knowledge and Attitudes items, demonstrating the item reliability. Confirmatory factorial analysis (*n* = 1450) showed that the selected model of Knowledge and Attitude constructs has adequate fit indicators and encompassed three factors that showed acceptable internal reliability: Knowledge (Cronbach’s alpha = 0.72), personal Attitudes towards tranquilizers (Cronbach’s alpha = 0.79) and Attitudes towards healthcare providers (Cronbach’s alpha = 0.65). The Arabic/French questionnaire was highly accepted, with a response rate of 95.72% and item non-response rate ≤3.6%. The availability of a cross-cultural adapted and multilingual validated questionnaire would stimulate research on tranquilizer misuse.

## 1. Introduction

The misuse of tranquilizers represents an emerging international public health concern with devastating impacts on the economy and society [1]. Tranquilizer misuse is associated with increased risk of road traffic accidents, poor management of comorbid diseases, deteriorated quality of life, elevated hospitalization and mortality rates and increased expenditures on health [2,3,4,5]. It is also associated with a high risk of dependence and can lead to illicit substance use [6].

Tranquilizers are misused when taken without medical prescription through sharing the drugs with someone else or using an old prescription [7,8]. Even when prescribed by a physician, tranquilizers can be misused if the patient fails to adhere to the instructions of use when skipping doses or taking the tranquilizers upon recall, using higher or lower dosages than prescribed, and/or extending or curtailing the treatment duration other than prescribed [3,4,9,10]. Storing leftover tranquilizers also represents an aspect of misuse of these drugs [9].

Tranquilizers are generally misused to improve sleep and reduce stress, for recreational motives, and to increment the effect of illicit substances [11,12,13]. The past decade witnessed an upward trend of tranquilizer misuse [1] and a simultaneous use of tranquilizers with other drugs such as opioids or cannabis [2]. In the United States, fatal and non-fatal drug overdoses from benzodiazepines taken alone or concurrently ingested with other drugs have substantially increased in recent years. The mortality rate attributed to overdose from concurrent use of tranquilizers with other drugs incremented 10 fold over the past decade [14], and related emergency department visits were stepped up by 90% [15]. In 2020, more than half of emergency department visits due to benzodiazepine overdose included females (51.5%), and around 21% were young adults [16]. In Europe, more than one-tenth of the population reported ever misusing tranquilizers and more than one-fifth of Europeans declared misusing sedatives in the past year [17]. Tranquilizer misuse is not only a challenge for developed countries, but also for developing countries [18,19,20,21], yet there is a shortage of information about the public health consequences of tranquilizer misuse in those countries. In Lebanon, 15% of university students reported misusing prescription drugs that were mainly obtained from parents and pharmacists [22,23].

Despite the alarming public health figures about the consequences of tranquilizer misuse, there has not been sufficient efforts from public health authorities to control the overprescribing and overuse of tranquilizers [24]. In addition, studies on the determinants of tranquilizer misuse have been scarce, with only a few studies examining the association of tranquilizers with sociodemographic, psychological and physical factors [1,25,26,27,28,29,30,31,32]. Determinants related to Knowledge and Attitudes towards tranquilizer misuse have also been understudied.

Knowledge, Attitudes and Practices are measured using a specific instrument called a Knowledge, Attitudes and Practices (KAP) questionnaire. Using KAP-modelled instruments, investigators can identify misconceptions in the population concerning a certain topic such as tranquilizer use, determine medically inappropriate attitudes and recognize medically inappropriate practices [33]. KAP questionnaires are therefore the cornerstone to assess the need for prevention programs aimed at improving certain health-related issues including the rational use of tranquilizers and to design and evaluate these programs [34,35].

So far, only two recent studies, one in a developing country and another in a developed country, have investigated the association between Knowledge and Attitudes with tranquilizer misuse Practices [36,37]. We recently developed and validated the first KAP instrument on tranquilizer use in Spanish language [38]. To maximize the usefulness of that tool across cultures and increase its applicability to non-Spanish speaking populations, and to stimulate the initiation of research on determinants of tranquilizer misuse worldwide, we aimed in the present study to adapt the questionnaire previously validated in Spanish and to validate it in a developing country, Lebanon, in French and Arabic, two official or co-official languages in more than 80 countries [39,40,41].

## 2. Materials and Methods

Study setting and population: The original KAP questionnaire on tranquilizer misuse was validated in Spain in Spanish language [38]. To validate the questionnaire in Lebanon, native multilingual researchers (BT, DAB and NM) translated it back and forth into Arabic and French (see Supplementary Materials Files S1 and S2). In Lebanon, the native and official language is Arabic, and French is considered the second language in the country [41]. French is spoken by half of the Lebanese population and taught in 70% of primary schools in Lebanon [41]. We adapted the sociodemographic characteristics section of the questionnaire to fit the Lebanese population by adding questions concerning total family income, employment status and education level of the spouse and access to healthcare.

The translated questionnaire was fully validated in the general adult population in Lebanon. Participants were parents of children recruited from schools in the capital, Beirut. The questionnaire was about the use of tranquilizers by the parents and not their children, yet we chose the schools to recruit the participants in order to ensure access to a sufficient number of adults in Lebanon. Eleven schools participated in the study. Participating schools informed the parents about the study objective, as well as about the expected questionnaire delivery and collection dates. The participants were aware that their participation was voluntary and anonymous. The schools chose the language of the questionnaire, Arabic or French, based on their knowledge of the parents’ characteristics.

Validation procedure: The procedure followed for the questionnaire validation in Lebanon was the same as that applied for the original questionnaire in Spain, and is described in detail elsewhere [38]. The questionnaire encompassed a total of 43 questions: 16 Knowledge and Attitude items that were answered by expressing the level of agreement on a given statement using a zero (strongly disagree) to ten (strongly agree) Likert scale, 11 Practice questions to be answered by selecting among a set of possible answers which investigated the source of tranquilizers (physician or others), adherence to the physician’s instructions in terms of timing, dosage and duration, as well as the action taken when extra tranquilizers were left unused, and 16 questions on sociodemographic characteristics.

Content validity: A panel of nine bilingual (Arabic and French) Lebanese experts who were specialized in pharmacy, medicine, or other health-related fields and who lived in Lebanon, evaluated each of the translated Arabic and French versions of the questionnaire [42,43]. Each expert examined every question in the questionnaire and scored it using one (lowest) to four (highest) Likert scale for its clarity and relevancy. Using the ratings of the experts, we calculated the following content validity indexes: item content validity index (I-CVI), scale content validity index average (S-CVI/Ave) and modified kappa (k*) [44,45].

Face validity: Following the questionnaire evaluation by the panel of experts, two researchers (NM and BT) reviewed the questionnaire for its clarity and completeness. Face validity is established when the instrument covers all the dimensions of the concept under study [43], i.e., Knowledge, Attitudes and Practices of tranquilizer use by the general adult population.

Pilot testing: Each version of the questionnaire, Arabic and French, was pilot tested in a sample of 20 socioeconomically different adults whose occupation was unrelated to health. The feedback of the participants on the clarity, format, ease of answering and length of the questionnaire was collected. Participants were also allowed to suggest modifications for improvement.

Reliability: Since Knowledge and Attitudes are stable variables over short durations, the reliability of Knowledge and Attitudes was tested through test–retest analysis. For this purpose, the questionnaire was distributed to 100 adults on two occasions within four-week time interval. Using data collected in the two test rounds, we estimated the intraclass correlation coefficient (ICC) relative to the average measure of the two-way mixed-effects model for each Knowledge and Attitude item [46]. An item was deemed reliable if its ICC value was >0.4 [47].

Construct validity: The validity of the Knowledge and Attitude construct of the questionnaire was explored using Confirmatory Factorial Analysis (CFA). Data for CFA analysis were collected from 1450 parents of school children. Knowledge and Attitude items were assigned to their corresponding factors following the pattern of the model of the questionnaire originally validated in Spain [38]. The items were allocated into three factors: (1) Knowledge, (2) Personal Attitudes towards tranquilizers and (3) Attitudes towards healthcare providers.

The factors were standardized by constraining them to a mean of 0 and a variance of 1. To improve the model fit of the questionnaire, we evaluated the standardized residual correlations between items and applied the modification indexes method [48,49]. Missing data were treated using the Full Information Maximum Likelihood method. We assessed the goodness of fit of the model by calculating the following statistics: Root Mean Squared Error Approximation (RSMEA, acceptable if <0.08), Comparative Fit Index (CFI, acceptable if ≥0.90), Tucker–Lewis Index (TLI, acceptable if ≥0.90) and Standardized Root Mean Square Residual (SRMR, acceptable if <0.08) [50]. In addition, we computed the chi-squared (*X*^2^) statistic value, Akaike Information Criterion (AIC), Bayesian Information Criterion (BIC) and sample size-adjusted BIC (aBIC). When comparing various models, the model that has the lowest *X*^2^, AIC, BIC and aBIC values is the one with the best quality [51].

Questionnaire overall reliability, acceptability and item response rate: The overall reliability of the questionnaire was evaluated by calculating Cronbach’s alpha index using data collected from 1450 parents of school children. Cronbach’s alpha index >0.6 was considered acceptable [52,53]. The acceptability of the questionnaire by the population was also explored by calculating the response rate, i.e., the percentage of answered questionnaires in the sample of 1450 adults. The acceptability of the items of the questionnaire was inspected by calculating the proportion of unanswered questions to the total number of returned questionnaires [54,55,56,57].

All statistical analyses were carried out using IBM SPSS 20.0 (SPSS Inc. Released 2020. SPSS for Windows, Version 20.0. Chicago). CFA was analyzed with R (version 4.0.0), and R package: lavaan (version 0.6–6)

## 3. Results

Content validity: The content validity indexes of each of the Arabic and French versions of the questionnaire which were calculated based on the experts’ evaluation showed good values: I-CVI ranged between 0.89 and 1.00, revealing the clarity, understandability and relatedness of the items to KAP about tranquilizer misuse; K* statistic was >0.89 and equal to I-CVI for all items, indicating that the agreement between the nine experts on item evaluation is improbably to have happened by chance; S-CVI/Ave was ≥0.95, thus establishing the content validity of the scale.

Face validity: The researchers who reviewed the final version of the Arabic and French questionnaires found that the items measured what they were intended to measure, thus establishing the face validity of the questionnaire.

Pilot testing: The questionnaire was completely answered by the 40 adults (20 in Arabic and 20 in French) who participated in the pilot testing. The participants showed satisfaction about the questionnaire format, design, length and ease of answering. They did not suggest any questionnaire amendment.

Test–retest analysis: Out of 100 participants, 91 answered the Arabic (*n* = 60) and French (*n* = 31) questionnaires on two occasions. ICC values were ≥0.54 for all Knowledge and Attitudes items in Arabic and ≥0.68 for those in French, thus establishing their reliability and capacity to generate reproducible data (Table 1).

Construct validity: We evaluated various models following theoretical and logical grounds and relying on the indications of the method of modification of indexes [48,49].

The initial model followed the structure of the model selected for the Spanish population and encompassed three factors: Knowledge; personal Attitudes towards tranquilizers; and Attitudes towards healthcare providers. Items Q5 to Q8 and Q10 were assigned to the “Knowledge” factor. Items Q1 to Q4 and Q11 were attributed to the “personal Attitudes towards tranquilizers” factor. Items Q9 and Q12 to Q16 were allocated to “Attitudes towards healthcare providers”. Item Q13 “If I believe that I need a tranquilizer and the doctor did not prescribe it, I will get it at the pharmacy without a prescription” did not significantly load on its respective factor and the model did not present adequate fit indicators (Table 2).

The initial model followed the structure of the model of the questionnaire validated in Spain. The final model included correlation of item residuals as suggested by the modification index method. χ^2^: Chi-square value; df: Degrees of Freedom; *p*: *p*-value (Chi-square); RSMEA: Root Mean Squared Error Approximation; CFI: Comparative Fit Index; TLI: Tucker–Lewis Index; AIC: Akaike Information Criterion, BIC: Bayesian Information Criterion; aBIC: sample size-adjusted BIC; SRMR: Standardized Root Mean Square Residual.

**Figure 1:**
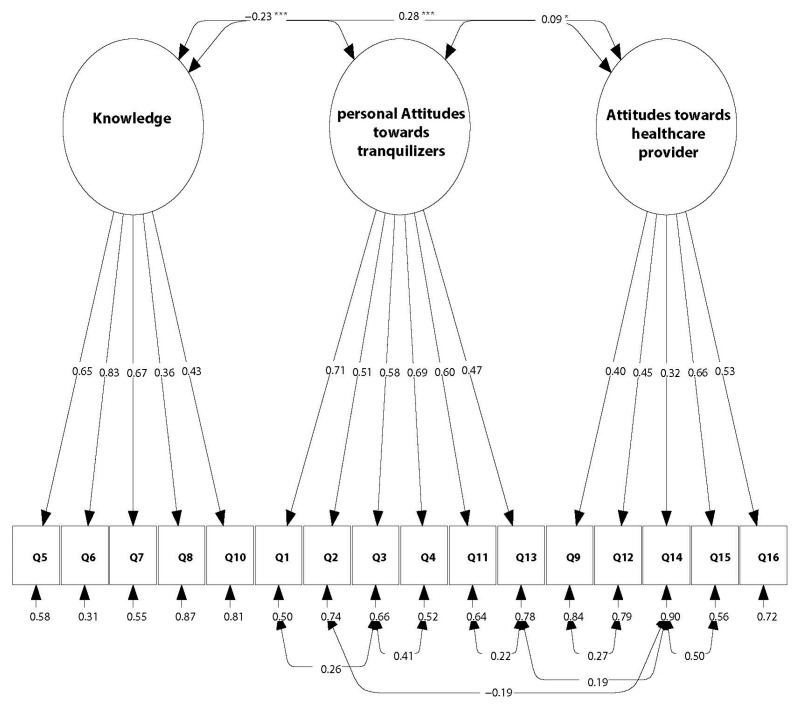
Chosen model for Knowledge and Attitude statements about tranquilizer in Lebanon. Figure represents the three factors of the model: Knowledge, personal Attitudes towards tranquilizers and Attitudes towards Healthcare providers, along with their corresponding standardized item loadings and item residuals. Double-sided arrows correspond to correlations between the variables. Single-headed arrows indicate the correlation of each item with its respective factor. The asterisks represent the level of significance: *** and * correspond to *p*-values < 0.0001 and < 0.01, respectively.

According to indications of the index modification method, item Q13 was moved to the “personal Attitudes towards tranquilizers” factor, and the residuals of various items were correlated. The final structure of the model is represented in Figure 1. In the final model, all items loaded significantly on their respective factors (Table 3). The residuals of the correlated items showed significant correlation (*p* < 0.001): Q1 and Q3 (r = 0.259), Q2 and Q14 (r = −0.194), Q3 and Q4 (r = 0.408), Q9 and Q12 (r = 0.268), Q11 and Q13 (r = 0.219), Q13 and Q14 (r = 0.194), and Q14 and Q15 (r = 0.498) (Figure 1). The fit indicators of the model were acceptable: RSMEA = 0.056, SRMR = 0.048, CFI = 0.925 and TLI = 0.905. The values of the X2 statistic, AIC, BIC and aBIC values of the final model, were the lowest among all the explored models (Table 2).

The Knowledge factor correlated negatively with the factor personal Attitudes towards tranquilizers (r = −0.227; *p* < 0.001), but it positively correlated with Attitudes towards healthcare providers (r = 0.284; *p* < 0.001). The factors “personal Attitudes towards tranquilizers” and “Attitudes towards healthcare providers” also showed a weak positive correlation (r = 0.089; *p* = 0.04; Figure 1).

Questionnaire internal reliability: The overall internal reliability of the questionnaire was acceptable with a Cronbach’s alpha value of 0.66. The internal reliability analysis per each of the three factors of the Knowledge and Attitude construct were also acceptable: “Knowledge” factor (Cronbach’s alpha = 0.72), “personal Attitudes towards tranquilizers” factor (Cronbach’s alpha = 0.79) and “Attitudes towards healthcare providers” factor (Cronbach’s alpha = 0.65).

Questionnaire acceptability: Out of 1450 distributed questionnaires, 1388 were completely or almost completely answered, representing a high response rate of 95.72% on the Arabic/French questionnaire by the general adult population in Lebanon. The item non-response rate was ≤ 3.6% for all items, establishing the high acceptability of the questions by the population.

## 4. Discussion

Tranquilizer misuse is a public health emergency that hit developed and developing countries and led to increasing morbidity and mortality rates. Intervention programs to improve the use of medicines rely on KAP-model questionnaires. In this study, we validated the first Arabic/French KAP questionnaire on tranquilizer use by the general adult population. The content and face validity of the questionnaire, as well as its reproducibility, construct validity and internal reliability, were established. High acceptability of the questionnaire by the general population was also demonstrated.

Despite the expansion of the tranquilizer misuse problem worldwide, research on determinants of Knowledge and Attitudes are scarce. Therefore, providing a cross-culturally validated questionnaire that can adapt to socioeconomic different countries and to various main languages (Arabic, French and Spanish) would: (1) stimulate the initiation of studies on tranquilizer misuse, (2) allow assessing the need for implementing intervention measures and (3) help design and evaluate prevention programs that better fit each population.

The face and content validity of the questionnaire showed the relatedness of the questions to the outcome and proved their clarity and comprehensiveness to tackle all the important aspects of tranquilizer misuse. The reproducibility of the questionnaire was demonstrated by the test–retest analysis which is essential to obtain high quality data. The validity of the construct was also established in the CFA analysis. The structure of the final model that best fits the data collected from the Lebanese population using the Arabic/French questionnaire was similar to that of the model chosen for the Spanish population using the previously validated Spanish version of the questionnaire [38]. In the model selected for the Lebanese population, the item Q13 *“If I believe that I need a tranquilizer and the doctor did not prescribe it, I will get it at the pharmacy without a prescription”* was allocated in the “personal Attitudes towards tranquilizers” factor unlike in the model defined for the Spanish population where this item belonged to the “Attitudes towards healthcare providers” factor. This modification can be explained by the fact that in Lebanon, tranquilizers are more accessible than in Spain. In Lebanon, the medical prescriptions are not computerized and individuals can obtain tranquilizers via old prescriptions, using multiple prescriptions from different physicians, and/or via prescriptions given for someone else. Studies also showed that in Lebanon, the use of a medication in the past is the most common motive for self-medication with that drug [58], and that tranquilizers can be obtained from family members as well as from pharmacists without prescription [22]. Moreover, the difficult socioeconomical situation that Lebanon has been experiencing over recent decades contributes to the misuse of tranquilizers, due to the association between social stressors and tranquilizer misuse [59,60]. Another contributor that favours the misuse of tranquilizers is the perception of the population of these drugs as safer and more socially accepted than illicit drugs [61,62].

The model selected for the Lebanese population involved a correlation between the residuals of various Knowledge and Attitude items, an expected observation in questionnaires that encompass more than one factor [63].

Our study is limited by the absence of an instrument superior to our questionnaire which could serve as a gold standard to which the performance of our questionnaire could be compared. In addition, some items loadings showed a low magnitude in the construction of their respective latent factors (i.e., item Q8 and Q14). This might affect the instrument convergent and differential validity. However, the low correlation between factors might indicate that latent factors are independent from each other. Future studies that use the proposed instrument should assess convergent and differential validity, and test the validation of the same questionnaire in other French or Arabic speaking countries to check to what extent the performance of this tool is dependent on the cultural construct more than on the language. Despite these limitations, the full validation of the questionnaire in two socioeconomically different populations and very divergent public health systems demonstrates the capacity of the questionnaire to reliably measure Knowledge, Attitudes and Practices towards tranquilizer use.

## 5. Conclusions

A KAP questionnaire on tranquilizer use by the general population was validated in Arabic and French. The validated Arabic/French version of the questionnaire will prove useful to initiate research on tranquilizer misuse in more than 80 countries and will help the corresponding authorities in the assessment of the need for measures to control the misuse of tranquilizers as well as in their implementation.

## Figures and Tables

**Table 1:** Test–retest reliability assessment of Knowledge and Attitudes items of the KAP questionnaire on tranquilizer use.

Knowledge and Attitude Statements	Arabic Version ICC (95% CI)	French Version ICC (95% CI)	Original Spanish Version [38] ICC (95% CI)
**Q1.** I would agree to take tranquilizers in order to sleep better	0.74 (0.57, 0.85)	0.86 (0.71, 0.93)	0.84 (0.77, 0.88)
**Q2.** If I feel better after a few days, I sometimes keep taking my tranquilizers even after completing the prescribed course of treatment	0.78 (0.62, 0.87)	0.69 (0.35, 0.85)	0.72 (0.60, 0.80)
**Q3.** I would take tranquilizers in order to enjoy myself with my family	0.76 (0.60, 0.89)	0.76 (0.50, 0.88)	0.77 (0.67, 0.83)
**Q4.** I would agree to take tranquilizers when I feel down and sad in order to work better	0.77 (0.61, 0.86)	0.77 (0.52, 0.89)	0.73 (0.62, 0.81)
**Q5.** Tranquilizers reduce people’s control over what they do	0.63 (0.38, 0.78)	0.68 (0.34, 0.85)	0.68 (0.55, 0.78)
**Q6.** People taking tranquilizers are at increased risk of traffic accidents	0.54 (0.23, 0.73)	0.77 (0.52, 0.89)	0.71 (0.59, 0.79)
**Q7.** Psychotropic drugs (such as tranquilizers) may affect children’s learning abilities when prescribed to them	0.70 (0.50, 0.82)	0.88 (0.75, 0.94)	0.71 (0.59, 0.80)
**Q8.** If I feel side effects during a course of treatment of tranquilizers, I should stop taking it as soon as possible	0.57 (0.28, 0.74)	0.94 (0.87, 0.97)	0.60 (0.43, 0.71)
**Q9.** I would take the tranquilizers according to the doctor’s instructions	0.66 (0.43, 0.80)	0.88 (0.75, 0.94)	0.77 (0.67, 0.84)
**Q10.** If tranquilizers are consumed in excess, they will not work when they are really needed	0.75 (0.59, 0.85)	0.82 (0.63, 0.91)	0.59 (0.42, 0.71)
**Q11.** I prefer to keep tranquilizers at home in case there is a need for them later	0.65 (0.42, 0.79)	0.92 (0.83, 0.96)	0.69 (0.56, 0.78)
**Q12.** I will trust the doctor’s decision if s/he decides to prescribe or not prescribe tranquilizers	0.66 (0.43, 0.70)	0.75 (0.47, 0.88)	0.82 (0.74, 0.87)
**Q13.** If I believe that I need a tranquilizer and the doctor did not prescribe it, I will get it at the pharmacy without a prescription	0.68 (0.47, 0.81)	0.88 (0.75, 0.94)	0.53 (0.34, 0.67)
**Q14.** I think that doctors often explain clearly to the patient the reasons for prescribing or not prescribing tranquilizers	0.67 (0.45, 0.81)	0.72 (0.43, 0.87)	0.65 (0.51, 0.75)
**Q15.** I think that doctors often explain clearly to the patient the instructions for the use of tranquilizers	0.82 (0.69, 0.89)	0.88 (0.75, 0.94)	0.63 (0.47, 0.74)
**Q16.** I thin that, when dispensing tranquilizers, the pharmacist tells the customer about the importance of correct therapeutic compliance/adherence	0.76 (0.59, 0.86)	0.78 (0.55, 0.90)	0.65 (0.51, 0.76)

ICC: intra-class correlation coefficient; CI: confidence interval.

**Table 2:** Comparison of the goodness of fit parameters between models of KAP questionnaire on tranquilizer use.

Indicator	Initial Model	Final Model
χ2	1078.304	499.393
df	101	94
*p*	<0.001	<0.001
RSMEA (90% CI)	0.083 (0.079, 0.088)	0.056 (0.051, 0.060)
CFI	0.820	0.925
TLI	0.786	0.905
AIC	102,837.055	102,272.144
BIC	103,104.365	102,576.144
aBIC	102,942.357	102,391.899
SRMR	0.064	0.048

**Table 3:** Factor loadings and standard errors from the three-factor model of the KAP questionnaire on tranquilizer use.

Item	Loading Estimate	Standard Error	*p*-Value	Standard Loading Estimate
**Knowledge**				
**Q5.** Tranquilizers reduce people’s control over what they do	1.247	0.042	<0.001	2.084
**Q6.** People taking tranquilizers are at increased risk of traffic accidents	1.363	0.040	<0.001	2.277
**Q7.** Psychotropic drugs (such as tranquilizers) may affect children’s learning abilities when prescribed to them	1.045	0.035	<0.001	1.745
**Q8.** If I feel side effects during a course of treatment of tranquilizers, I should stop taking it as soon as possible	0.660	0.047	<0.001	1.103
**Q10.** If tranquilizers are consumed in excess, they will not work when they are really needed	0.684	0.040	<0.001	1.143
**Personal Attitudes towards tranquilizers**				
**Q1.** I would agree to take tranquilizers in order to sleep better	1.212	0.041	<0.001	1.930
**Q2.** If I feel better after a few days, I sometimes keep taking my tranquilizers even after completing the prescribed course of treatment	0.763	0.038	<0.001	1.215
**Q3.** I would take tranquilizers in order to enjoy myself with my family	0.900	0.040	<0.001	1.434
**Q4.** I would agree to take tranquilizers when I feel down and sad in order to work better	1.200	0.040	<0.001	1.911
**Q11.** I prefer to keep tranquilizers at home in case there is a need for them later	1.255	0.050	<0.001	1.998
**Q13.** If I believe that I need a tranquilizer and the doctor did not prescribe it, I will get it at the pharmacy without a prescription	0.669	0.038	<0.001	1.066
**Attitudes towards healthcare providers**				
**Q9.** I would take the tranquilizers according to the doctor’s instructions	0.727	0.059	<0.001	1.046
**Q12.** I will trust the doctor’s decision if s/he decides to prescribe or not prescribe tranquilizers	0.994	0.072	<0.001	1.432
**Q14.** I think that doctors often explain clearly to the patient the reasons for prescribing or not prescribing tranquilizers	0.795	0.081	<0.001	1.144
**Q15.** I think that doctors often explain clearly to the patient the instructions for the use of tranquilizers	1.349	0.071	<0.001	1.943
**Q16.** I think that, when dispensing tranquilizers, the pharmacist tells the customer about the importance of correct therapeutic compliance/adherence	1.136	0.079	<0.001	1.635

## Data Availability

The data generated and analyzed in this study can be made available upon reasonable request from the corresponding author.

## Supplementary Materials

The following are available online at https://www.mdpi.com/article/10.3390/ijerph182111144/s1, File S1: Arabic version of the validated KAP questionnaire, File S2: French version of the validated KAP questionnaire.
