# Supplementary material for: Validation of the Arabic and French Versions of a Knowledge, Attitudes and Practices (KAP) Questionnaire on Tranquilizer Misuse
Source: Int J Environ Res Public Health. 2021 Oct 23;18(21):11144. doi: 10.3390/ijerph182111144 (PMC8582760; doi:10.3390/ijerph182111144)
Supplement: Supplementary file 1 [file ijerph-18-11144-s001.zip › ijerph-1383397-supplementary/S1.pdf]

USC UNIVERSIDADE DE SANTIAGO DE COMPOSTELA — FACULTADE DE MEDICINA E ODONTOLOXÍA

AUST

المَعرفَة والإعتِقَاد وَإستِخدَام المُهدِّئَات

رمز الإستبيان: ____________ التَّارِيخ: ________________

يُرجَى الإجَابَة عَلَى الأسئِلَة التَّالِيَة حَوْلَ *المُهَدِّئَات* (أدويه تتَضَمَّن: المنوّم أو حبوب الأعصاب، مثلاً: ألبرازولام، برومازيبام، ديازيبام، أمبين، زاناكس، فاليوم، إلى آخره)؟

على مقياس من "⓪" (صفر) إلى "⑩"(عشر) نقاط، إختر رقم يعبر عن درجة موافقتك على العبارات التَّالية، بحيث أن الرقم "⓪" (صفر) يعني إنّك ترفض كُليّاً والرقم "⑩" (عشرة) يعني إنّك توافق تماماً

يُرجى تقييم العبارات التَّالية **بصرف النَّظر** عمّا إذا كُنت تستخدم *المُهَدِّئَات* **أم لا**

مثلاً: مُمارسة ا لرِّياضة تُفيد الصِّحَّة ⓪ ① ② ③ ④ ⑤ ⑥ ⑦ ● ⑨ ⑩

	**لا أوافِق** →									← **أوافِق**
	0	1	2	3	4	5	6	7	8	9	10

١) من الممكن أن أوافق على إستعمال *المُهَدِّئَات* لكي أنام بشكل أفضل ⓪ ① ② ③ ④ ⑤ ⑥ ⑦ ⑧ ⑨ ⑩

٢) إذا شعرت بتحسن بعد بضعة أيّام أَتابع إستعمال *المُهَدِّئَات* حتى بعد إنتهاء فترة العلاج المقرَّرة ⓪ ① ② ③ ④ ⑤ ⑥ ⑦ ⑧ ⑨ ⑩

٣) من الممكن أن أستعمل *المُهَدِّئَات* لأستمتع أكثر مع عائلتي ⓪ ① ② ③ ④ ⑤ ⑥ ⑦ ⑧ ⑨ ⑩

٤) من الممكن أن أوافق على إستعمال *المُهَدِّئَات* إذا كنت محبط وحزين، لكي أعمل بشكلٍ أفضل ⓪ ① ② ③ ④ ⑤ ⑥ ⑦ ⑧ ⑨ ⑩

٥) *المُهَدِّئَات* تخفف من قدرة النَّاس على التّحكم بتصرّفاتهم ⓪ ① ② ③ ④ ⑤ ⑥ ⑦ ⑧ ⑨ ⑩

٦) الأشخاص الذين يتناولون *المُهَدِّئَات* معرَّضين بشكل أكبر لخطر حدوث حوادث السَّير ⓪ ① ② ③ ④ ⑤ ⑥ ⑦ ⑧ ⑨ ⑩

٧) قد يكون للأدوية التي توثّر على الأعصاب مثل *المُهَدِّئَات* تأثيرعلى قدرات الأطفال التعليميّة عند وصفها لهم ⓪ ① ② ③ ④ ⑤ ⑥ ⑦ ⑧ ⑨ ⑩

٨) إذا شعرت بآثار جانبيّة خلال فترة العلاج *بالمهُ دِّ ئَا ت* يجب أن أتوقف عن إستعماله في أقرب وقت ممكن ⓪ ① ② ③ ④ ⑤ ⑥ ⑦ ⑧ ⑨ ⑩

٩) أستعمل المُهَدِّئَات حسب تعليمات الطّبيب ⓪ ① ② ③ ④ ⑤ ⑥ ⑦ ⑧ ⑨ ⑩

١٠) إذا تمّ إستهلاك المُهَدِّئَات بكثرةٍ، فلن تنفع عند الحاجة إليها حقًاً ⓪ ① ② ③ ④ ⑤ ⑥ ⑦ ⑧ ⑨ ⑩

١١) أُفضّل أن أُبقي مهُدِّئَات إضافيّة في المنزل، في حال الحاجة إليها لاحقاً ⓪ ① ② ③ ④ ⑤ ⑥ ⑦ ⑧ ⑨ ⑩

١٢) أثق بقرارالطبيب سواء قرر وصف أو عدم وصف المُهَدِّئَات ⓪ ① ② ③ ④ ⑤ ⑥ ⑦ ⑧ ⑨ ⑩

١٣) إذا كنت أعتقد أنّني بحاجة إلى المُهَدِّئَاتَ ولم يصفها الطبيب، سأحصل عليها من الصَّيْدَلِيَّة بدون وصفة ⓪ ① ② ③ ④ ⑤ ⑥ ⑦ ⑧ ⑨ ⑩

١٤) غَالِبًا مَا يَشرَح الأَطبَّاء بِوضوح للمريض أَسباب وصف أَو عدم وصف المُهَدِّئَات ⓪ ① ② ③ ④ ⑤ ⑥ ⑦ ⑧ ⑨ ⑩

١٥) غَالِبًا مَا يَشرَح الأَطبَّاء بِوضوح للمريض كيفية إستخدام المُهَدِّئَات ⓪ ① ② ③ ④ ⑤ ⑥ ⑦ ⑧ ⑨ ⑩

١٦) عند شراء المُهَدِّئَات، يُبلغك الصَّيْدَلانيّ عن أهميّة التَّقيُّد الصَّحيح بالعلاج ⓪ ① ② ③ ④ ⑤ ⑥ ⑦ ⑧ ⑨ ⑩

١٧) في الشَّهر الماضي، هل إستعملت المُهَدِّئَات؟ ◯ نعم (رجاءً إنتقل إلى االسُّؤال ١٨) ◯ لا (رجاءً إنتقل إلى االسُّؤال ٢٨)

أَجِب على الأَسْئِلَةِ التَّالِيَة عن إستهلاكك من *المُهَدِّئَات* في الشَّهر الماضي

١٨) كم كانت مُدَّة آخِرعلاج بالمُهَدِّئَات؟

عدد الأَيَّام: ☐☐ عدد الأشهر: ☐☐ عدد السِّنين: ☐☐

١٩) من الَّذي وصف لك أو نصحك بإستخدام المُهَدِّئَات؟ (بإمكانك إختيار أكثر من إجابة)

◯ الطَّبيب ◯ الأَصْدِقَاء

◯ العائلة ◯ الصَّيْدَلانيّ

◯ كانَ عندي في المنزل

٢٠) في آخر مَرَّة كان عليك أن تتناول المُهَدِّئَات، هل أكملت كُلّ دورة العلاج؟

◯ نعم ◯ كلا

٢١) ماذا فعلت بالمُهَدِّئَات الّتي تُركت دون إستعمال؟ (بإمكانك إختيار أكثر من إجابة)

◯ إحتفظت بها لكي أَستَعمِلُهَا عندما أمرض في المَرَّة القادمة

◯ رميتها في الزباله أو في مياه الصَّرف الصِّحِّيّ

◯ أُعطيتها لأحد

◯ لم يتبقى لديّ أي من المُهَدِّئَات

٢٢) في آخر مَرَّة كان عليك أن تتناول المُهَدِّئَات، هل نسيت أن تأخذ أي من الجرعات؟

◯ أبداً (رجاءً إنتقل إلى االسُّؤال ٢٤)

◯ أحياناً (رجاءً إنتقل إلى االسُّؤال ٢٣)

◯ في كثير من الأحيان (رجاءً إنتقل إلى السُّؤال ٢٣)

٢٣) ماذا فعلت عندما تخطَّيت/لم تتناول جُرْ عَة من المُهَدِّئَات؟

◯ تابعت الجُرعات التَّالِيَة بشكل طبيعي

◯ ضاعفت أو زدت الجُرعة الثانيّة

◯ تناولتها عندما تذكرت

٢٤) في آخر مَرَّة كان عليك أن تتناول المُهَدِّئَات، هل قُمت بتغيير عيار الجُرعة من تِلْقَاء نفسك (بِدُون إسْتِشَارَةٍ طِبِّيَّةٍ)؟

◯ أبداً (رجاءً إنتقل إلى االسُّؤال ٢٨)

◯ أحياناً (رجاءً إنتقل إلى االسُّؤال ٢٥)

◯ في كثيرٍ من الأحيان (رجاءً إنتقل إلى االسُّؤال ٢٥)

٢٥) ماذا فعلت عندما غيَّرتُ عيار جُرعة المُهَدِّئَات من تِلْقَا ء نفسك (بِدُون إسْتِشَارَةٍ طِبِّيَّةٍ)؟

◯ تناولت منها أكثر (رجاءً إنتقل إلى االسُّؤال ٢٦ ومن وثمَّ إلى السُّؤال ٢٨)

◯ تناولت منها أقل (رجاءً إنتقل إلى االسُّؤال ٢٧)

◯ أحياناً تناولت منها أكثر وأحياناً أخرى أقل (رجاءً أجب على السُّؤالين ٢٦ و٢٧)

٢٦) لماذا قُمت بزيادة عيار جُرعة المُهَدِّئَات من تِلْقَاء نفسك (بِدُون إسْتِشَارَةٍ طِبِّيَّةٍ)؟ (بإمكانك إختيار أكثر من إجابة)

◯ نسيت أن أتناول الجُرعة السَّابقة

◯ شعرت بأنَّني مريضٌ جدًّا

◯ لم أشعر بتحسُّن ملحوظ

◯ تحسَّنت ولكن أردت أن أتحسَّن أكثر

◯ كُنتُ أمر بمرحلةٍ عصبيةٍ (ضغوطات، مشاكل،إلى آخره)

٢٧) لماذا قُمت بتخفيض عيار جُرعة المُهَدِّئَات من تِلْقَاء نفسك (بِدُون إسْتِشَارَةٍ طِبِّيَّةٍ)؟ (بإمكانك إختيار أكثر من إجابة)

◯ كُنتُ قلقاً من آثار الدواء الجانبيّة

◯ كُنتُ متعباً ونسيت

◯ شعرت بأنّني آخذ الكثير من الدّواء

◯ كُنتُ على عجلة ونسيت

◯ لا أحب تناول الأَدْوِيَة في اللَّيل

◯ شعرت بتحسُّن

المَعرفَة والإعتِقَاد وَاِستِخدَام المُهَدِّئَات

رمز الإستبيان: __________ التَّارِيخ: __________

الخَصَائِص الدِّيمُوغْرَافِيَّة العَامَّة

٢٨) حدِّد جِنسك ◯ ذكر ◯ أنثى

٢٩) ما هو تاريخ ميلادك؟ (يُرجى كتابة الأرقام المقابلة في الفراغات أدناه)

☐☐ ☐☐ ☐☐☐☐

اليَوْم الشَّهْر السَّنَة

٣٠) ما هو أعلى مستوى تعليميّ لديك؟

◯ لم أدخل إلى المدرسة

◯ إبتدائيّ (الصّف الأول إلى الصّف السادس)

◯ مُتَوَسِّط (الصف السَّابع إلى الصف التَّاسِع)

◯ ثانويّ (الصف العَاشِر إلى الصف الثَّانِي عَشْر)

◯ جامعة

٣١) هل تعمل حاليّاً؟ ◯ نعم ◯ كلا

٣٢) ماذا يُغَطّي تَأْمِينُك الصِّحّيّ؟ (بإمكانك إختيار أكثر من إجابة)

◯ ليس لديّ تأمين صِحِّيّ

◯ التَّكلفة أو جزء من تكلفة الإستشارة الطبيَّة

◯ التّكلفة أو جزء من تكلفة الأدويَّة

٣٣) يُرجى الإشارة إلى وضعك العائلي؟

◯ متزوج/متزوجة (رجاءً إنتقل إلى االسُّؤال رقم ٣٤)

◯ مطلّق/مطلّقة (رجاءً إنتقل إلى االسُّؤال رقم ٣٦)

◯ أرمل/أرملة (رجاءً إنتقل إلى االسُّؤال رقم ٣٦)

◯ منفصل/منفصلة عن الزوجة/الزوج ولكن لستما مطلقين (رجاءً إنتقل إلى االسُّؤال رقم ٣٤)

٣٤) ما هو أعلى مستوى تعليمي لزوجك/لزوجتك؟

◯ لم يدخل/تدخل إلى المدرسة

◯ إبتدائيّ (الصف الأول إلى الصف السادس)

◯ مُتوسِّط (الصف السَّابع إلى الصف التَّاسِع)

◯ ثانويّ (الصف العَاشِر إلى الصف الثَّانِي عَشْر)

◯ جامعة

٣٥) هل زوجك/زوجتك يعمل/تعمل حاليّاً؟ ◯ نعم ◯ كلا

٣٦) ما هو عدد الأفراد الذين يعيشون في منزلك، بما في ذلك أنت؟

◯ ٢ ◯ ٣- ٤ ◯ ٥- ٦ ◯ أكثر من ٦

٣٧) هل توجد أيَّة مراكز رعاية صِحِّيَّة (مثلًا مستوصفات أو مستشفيات)، موجودة بالقرب من منطقة سكنك؟

◯ نعم ◯ كلا

٣٨) ما هو مجموع دخل الأسرة الشّهري (مجموع الدخل المُكتسب من جميع الأفراد الذين يعيشون في منزلك)؟

◯ أقل من ٥٠٠ دولارٍ أمريكيٍّ في الشّهر

◯ بين ٥٠٠ و٩٩٩ دولارٍ أمريكيٍّ في الشّهر

◯ بين ١٠٠٠ و ١٤٩٩ دولارٍ أمريكيٍّ في الشّهر

◯ بين ١٥٠٠ و ١٩٩٩ دولارٍ أمريكيٍّ في الشّهر

◯ بين ٢٠٠٠ و ٢٥٠٠ دولارٍ أمريكيٍّ في الشّهر

◯ أكثر من ٢٥٠٠ دولارٍ أمريكيٍّ في الشّهر

٣٩) هل تستشير الطّبيب عندما تكون مريضًا؟

◯ أَبَدًا ◯ نَادِرا

◯ أَحْيَانًا ◯ دَائِمًا (رجاءً إنتقل إلى االسُّؤال رقم ٤١)

٤٠) ما هو سبب عدم إستشارتك للطَّبيب بشكلٍ دائمٍ؟ (بإمكانك إختيار أكثر من إجابة)

◯ لا حاجة للطّبيب

◯ الخوف من الطّبيب

◯ عدم توفر المال الكافي

◯ عدم توفر الوقت الكافي لزيارة الطَّبيب

◯ فترة الإنتظار الطويلة لِيَتِمَّ فحص المريض من قبل الأطبَّاء

◯ عدم وجود عيادة بالقرب من منزلي

٤١) هل سبق لك أن تَلَقَّيْتَ وَصْفَة طِبِّيَّةً عبر الهاتف؟

◯ نعم ◯ كلا

٤٢) هل تتناول الكُحول أو المشروبات الروحيّة ؟

◯ نعم (رجاءً إنتقل إلى االسُّؤال رقم ٤٣)

◯ كلا (شكرًا لك! لقد أكملت الإجابة على الإستبيان)

٤٣) كم مرة تشرب الكُحول أو المشروبات الروحيّة؟

◯ في المُناسبات الخاصَّة ◯ ٣ - ٥ مرات في الأسبوع

◯ مَرَّة واحدة في الأسبوع ◯ يوميًّا

◯ في نهاية الأسبوع فقط

شكرًا جزيلاً لوقتكم وتعاونكم!
