# Supplementary material for: Validation of the Arabic and French Versions of a Knowledge, Attitudes and Practices (KAP) Questionnaire on Tranquilizer Misuse
Source: Int J Environ Res Public Health. 2021 Oct 23;18(21):11144. doi: 10.3390/ijerph182111144 (PMC8582760; doi:10.3390/ijerph182111144)
Supplement: Supplementary file 1 [file ijerph-18-11144-s001.zip › ijerph-1383397-supplementary/S2.pdf]

USC FACULTADE DE MEDICINA E ODONTOLOXÍA
UNIVERSIDADE DE SANTIAGO DE COMPOSTELA

AUST

CONNAISSANCES, CROYANCES ET UTILISATION DE TRANQUILLISANTS

Code du questionnaire:________________ Date:________________________

Veuillez répondre aux questions suivantes sur les *TRANQUILLISANTS (médicaments incluant somnifères ou calmants: Alprazolam, Bromazepam, Diazepam, Ambien, Xanax, Valium, etc.)*?

Sur une échelle de 0 à 10 points, sélectionnez un chiffre représentant votre niveau d'accord avec les affirmations ci-dessous. "0" (zéro) veut dire que vous n'êtes pas du tout d'accord et "10" représente un accord maximum.

Veuillez indiquer votre niveau d'accord **QUE VOUS** utilisiez des *tranquillisants* **OU PAS**

Exemple: Faire du sport est bénéfique pour la santé 0 1 2 3 4 5 6 7 ● 9 10

	← Pas d'accord — D'accord →
1. **J'accepterais de prendre des *tranquillisants* pour mieux dormir**	0 1 2 3 4 5 6 7 8 9 10
2. **Si je me sens mieux après quelques jours, je continue parfois de prendre des *tranquillisants* même après avoir fini le traitement prescrit**	0 1 2 3 4 5 6 7 8 9 10
3. **J'accepterais de prendre des *tranquillisants* pour pouvoir profiter du temps passé avec ma famille**	0 1 2 3 4 5 6 7 8 9 10
4. **J'accepterais de prendre des *tranquillisants* quand je suis abattu(e) et triste pour pouvoir mieux travailler**	0 1 2 3 4 5 6 7 8 9 10
5. **Les *tranquillisants* diminuent le contrôle des personnes sur ce qu'elles font**	0 1 2 3 4 5 6 7 8 9 10
6. **Les personnes qui prennent des *tranquillisants* ont un plus grand risque d'avoir un accident de la route**	0 1 2 3 4 5 6 7 8 9 10
7. **Lorsqu'ils sont prescrits à des enfants, les médicaments psychotropes (comme par exemple les *tranquillisants*), peuvent affecter leur capacité d'apprentissage**	0 1 2 3 4 5 6 7 8 9 10
8. **Si je ressens des effets secondaires durant un traitement de tranquillisants, je dois arrêter de prendre le médicament dès que possible**	0 1 2 3 4 5 6 7 8 9 10
9. **Je prends les *tranquillisants* en suivant les instructions du médecin**	0 1 2 3 4 5 6 7 8 9 10
10. **Quand les *tranquillisants* sont consommés de façon excessive, ils n'auront plus d'effet au moment où l'on en a vraiment besoin**	0 1 2 3 4 5 6 7 8 9 10
11. **Je préfère garder des *tranquillisants* chez moi au cas où j'en aurais besoin plus tard**	0 1 2 3 4 5 6 7 8 9 10
12. **Je fais confiance au médecin s'il /si elle décide de prescrire ou pas des *tranquillisants***	0 1 2 3 4 5 6 7 8 9 10
13. **Si je pense que j'ai besoin d'un *tranquillisant* et que le médecin ne me le prescrit pas, je vais à la pharmacie pour me le procurer sans ordonnance**	0 1 2 3 4 5 6 7 8 9 10
14. **Les médecins expliquent souvent clairement au patient les raisons de prescrire ou de ne pas prescrire des tranquillisants**	0 1 2 3 4 5 6 7 8 9 10
15. **Les médecins expliquent souvent clairement au patient les instructions d'utilisation des *tranquillisants***	0 1 2 3 4 5 6 7 8 9 10
16. **Quand le patient achète un tranquillisant, le pharmacien lui explique l'importance d'une bonne adhésion thérapeutique**	0 1 2 3 4 5 6 7 8 9 10

17. Au cours du mois dernier, avez-vous pris des *tranquillisants?*

○ Oui (passer à la question 18) ○ Non (passer à la question 28)

Veuillez répondre aux questions suivantes sur votre consommation des <u>TRANQUILLISANTS AU COURS DU MOIS DERNIER</u>

18. Quelle a été la durée de votre dernier traitement aux *tranquillisants*?

Nombre de jours ☐☐
Nombre de mois ☐☐
Nombre d'années ☐☐

19. Qui vous a prescrit ou recommandé les *tranquillisants*? (Vous pouvez choisir plusieurs réponses)

○ Le médecin ○ Des amis
○ La famille ○ Le pharmacien
○ Je les avais chez moi

20. Avez-vous pris jusqu'au bout votre dernier traitement aux *tranquillisants*?

○ Oui ○ Non

21. Qu'avez-vous fait des tranquillisants que vous n'avez pas utilisés? (Vous pouvez choisir plusieurs réponses)

○ Je les ai gardés pour les utiliser la prochaine fois que je suis malade
○ Je m'en suis débarrassé(e) dans la poubelle ou les eaux usées
○ Je les ai donnés à quelqu'un d'autre
○ Il ne me restait plus des tranquillisants

22. Au cours de votre dernier traitement aux tranquillisants, vous est-il arrivé d'oublier de les prendre?

○ Jamais (passer à la question 24)
○ Parfois (passer à la question 23)
○ Souvent (passer à la question 23)

23. Qu'avez-vous fait quand vous avez oublié de prendre une dose de vos tranquillisants?

○ J'ai continué de prendre les doses suivantes normalement
○ J'ai doublé/augmenté la dose suivante
○ Je l'ai prise dès que je m'en suis souvenu(e)

24. Au cours de votre dernier traitement aux tranquillisants, avez-vous changé la dose sur votre propre décision (sans avis médical)**?**

○ Jamais (passer à la question 28)
○ Parfois (passer à la question 25)
○ Souvent (passer à la question 25)

25. Comment avez-vous changé vous-même votre dose de tranquillisants (sans avis médical)**?**

○ J'en ai pris plus (passer à la question 26 puis à la question 28)
○ J'en ai pris moins (passer à la question 27)
○ Parfois j'en ai pris plus et parfois moins (passer à la question 26 puis 27)

26. Pourquoi avez-vous vous-même décidé d'augmenter votre dose de tranquillisants (sans avis médical)**?** (Vous pouvez choisir plusieurs réponses)

○ J'ai oublié de prendre la dose précédente
○ Je me suis senti(e) très malade
○ Je n'ai pas senti de réèlle amélioration
○ J'ai senti une amélioration mais voulais me sentir encore mieux
○ Je vivais une période stressante (stress, problèmes, etc…)

27. Pourquoi avez-vous vous-même choisi de réduire votre dose de *tranquillisants* (sans avis médical)**?** (vous pouvez choisir plusieurs réponses)

○ J'avais peur des effets secondaires du médicament
○ J'étais fatigué(e) et j'ai oublié
○ J'ai pensé que j'en prenais trop
○ J'étais pressé(e) et j'ai oublié
○ Je n'aime pas prendre de médicaments la nuit
○ Je me sentais mieux

Caractéristiques Démographiques Générales

28. Indiquez votre sexe ○ Homme ○ Femme

29. Quelle est votre date de naissance? (Veuillez inscrire les chiffres dans les espaces alloués)

☐☐ ☐☐ ☐☐☐☐
Jour **Mois** **Année**

30. Quel est votre niveau d'éducation académique.

○ Je n'ai pas été à l'école
○ Elémentaire/ Primaire (maternelle jusqu'à la 6ème)
○ Complémentaire (5ème à la 3ème)
○ Secondaire (2nde à la terminale)
○ Universitaire

31. Actuellement, avez-vous une activité professionnelle?

○ Oui ○ Non

32. Qu'est-ce qui est couvert par votre assurance santé? (Vous pouvez choisir plus d'une réponse).

○ Je n'ai pas d'assurance santé
○ Le coût ou une partie des coûts des consultations médicales
○ Le coût ou une partie des coûts de mes médicaments

33. Veuillez indiquer votre statut marital:

○ Marié(e) (passer à la question 34)
○ Divorcé(e) (passer à la question 36)
○ Veuf/Veuve (passer à la question 36)
○ Séparé(e) de mon épouse/époux, mais non divorcé(e) (passer à la question 34)

34. Quel est le niveau d'éducation académique de votre époux/épouse?

○ Il/Elle n'a pas été à l'école
○ Elémentaire/ Primaire (maternelle jusqu'à la 6ème)
○ Complémentaire (5ème à la 3ème)
○ Secondaire (2nde à la terminale)
○ Universitaire

35. Actuellement, votre époux/épouse travaille-t-il/elle?

○ Oui ○ Non

36. Combien de personnes vivent sous votre toit, inclus vous-même?

○ 2 ○ 3 – 4 ○ 5 – 6 ○ Plus de 6

37. Y a-t-il des instituts de santé, tels que des dispensaires ou hôpitaux, situés près de votre lieu de résidence?

○ Oui ○ Non

38. Quel est le total des revenus familiaux (quel est le revenu total perçu par tous les membres de la famille vivant sous le même toit)?

○ Moins de 500US$ par mois
○ Entre 500 et 999US$ par mois
○ Entre 1000 et 1499US$ par mois
○ Entre 1500 et 1999US$ par mois
○ Entre 2000 et 2500US$ par mois
○ Plus de 2500US$ par mois

39. Consultez-vous un médecin lorsque vous êtes malade?

○ Jamais ○ Rarement
○ Parfois ○ Toujours (passer à la question 41)

40. Quelle est la raison pour laquelle vous ne consultez pas toujours un médecin? (Vous pouvez choisir plus d'une réponse).

○ Je n'ai pas besoin de médecin
○ J'ai peur des médecins
○ Par manque d´argent
○ Je n'ai pas le temps de le consulter
○ L'attente avant d'être examiné(e) est longue
○ La clinique est située loin de ma maison

41. Avez-vous déjà reçu une prescription médicale par téléphone?

○ Oui ○ Non

42. Buvez-vous de l'alcool?

○ Oui (passer à la question 43)
○ Non (Merci! Vous avez fini de répondre au questionnaire)

43. A quelle fréquence consommez-vous de l'alcool?

○ Aux occasions spéciales
○ Une fois par semaine
○ Les weekends seulement
○ 3 à 5 fois par semaine
○ Tous les jours

Merci d'avoir participé à cette étude !
